# Calcium Pyrophosphate Crystal Formation and Deposition: Where Do we Stand and What Does the Future hold?

**DOI:** 10.1007/s11926-024-01161-w

**Published:** 2024-08-01

**Authors:** Silvia Sirotti, Anna Scanu, Tristan Pascart, Tom Niessink, Paola Maroni, Giovanni Lombardi, Georgios Filippou

**Affiliations:** 1 Rheumatology Department, IRCCS Galeazzi – Sant’Ambrogio Hospital, Milan, Italy; 2 Department of Women’s and Children’s Health, University of Padova, Padua, Italy; 3 Department of Neuroscience, University of Padova, Padua, Italy; 4 Department of Rheumatology, ETHICS Laboratory, Saint-Philibert Hospital, Lille Catholic University, Lille, France; 5 Personalized Diagnostics and Therapeutics, Technical Medicine Centre, University of Twente, Enschede, the Netherlands; 6 Department of Rheumatology, VieCuri Medical Centre, Venlo, the Netherlands; 7 Laboratory of Experimental Biochemistry and Molecular Biology, IRCCS Galeazzi – Sant’Ambrogio Hospital, Milan, Italy; 8 Department of Athletics, Strength and Conditioning, Poznań University of Physical Education, Poznań, Poland; 9 Department of Biomedical and Clinical Sciences, University of Milan, Milan, Italy

**Keywords:** CPPD, Chondrocalcinosis, Pathogenesis, Imaging, Basic science

## Abstract

**Purpose of the review:**

Although calcium pyrophosphate deposition (CPPD) has been known since the 1960s, our understanding of its pathogenesis remains rudimentary. This review aims to illustrate the known mechanisms underlying calcium pyrophosphate (CPP) crystal formation and deposition and explore future directions in research. By examining various perspectives, from basic research to clinical and imaging assessments, as well as new emerging methodologies, we can establish a starting point for a deeper understanding of CPPD pathogenesis.

**Recent Findings:**

Recent years have seen significant advances in CPPD research, particularly in the clinical field with the development of the 2023 ACR/EULAR classification criteria for CPPD disease, and in imaging with the introduction of the OMERACT ultrasonographic definitions and scoring system. However, progress in basic research has been slower. New laboratory approaches, such as Raman spectroscopy and omics sciences, offer promising insights that may help piece together the puzzle of CPPD.

**Summary:**

CPPD is a common yet understudied condition. As the population ages and CPPD becomes more prevalent, there is an urgent need to better understand the disease and the mechanisms involved in crystal formation and deposition, in order to improve diagnosis and therapeutic approaches.

## Introduction

Understanding calcium pyrophosphate (CPP) crystal formation and deposition remains an area ripe for exploration, with numerous aspects awaiting clarification [1–3]. Calcium pyrophosphate deposition (CPPD) is prevalent, especially among older people, and encompasses a broad spectrum of clinical presentations, ranging from asymptomatic deposition to acute or chronic arthritis with a significant impact on patients' lives [4].

Our current comprehension of CPP crystal formation derives from studies on cellular and extracellular mechanisms, ion imbalances, and investigations into genetic conditions that predispose to CPPD. Imaging studies, particularly conventional radiography (CR) and ultrasound (US) have primarily elucidated the joints and structures most frequently affected by CPP crystal deposition, along with their association with other conditions and the prevalence of depositions within selected populations. Newer modalities, such as dual-energy conventional tomography (DECT) and Raman spectroscopy are gaining prominence in this field, and hold promise for providing novel insight into this disorder.

This review focuses on illustrating the mechanisms underlying CPP crystal formation and deposition, examining different perspectives that collectively could hopefully enhance a better understanding of CPPD pathogenesis.

## Lessons from the Lab

### Cell changes in CPPD

Although the exact mechanisms that lead to the formation of CPP crystals are not fully understood, various cell types including chondrocytes, synoviocytes, as well as inflammatory and bone cells are differently involved in the production of crystals and in the inflammatory response. Much of the research on the interaction between joint cells and CPP crystals stems from studies conducted in patients with osteoarthritis (OA) [5].

**Fig. 1:**
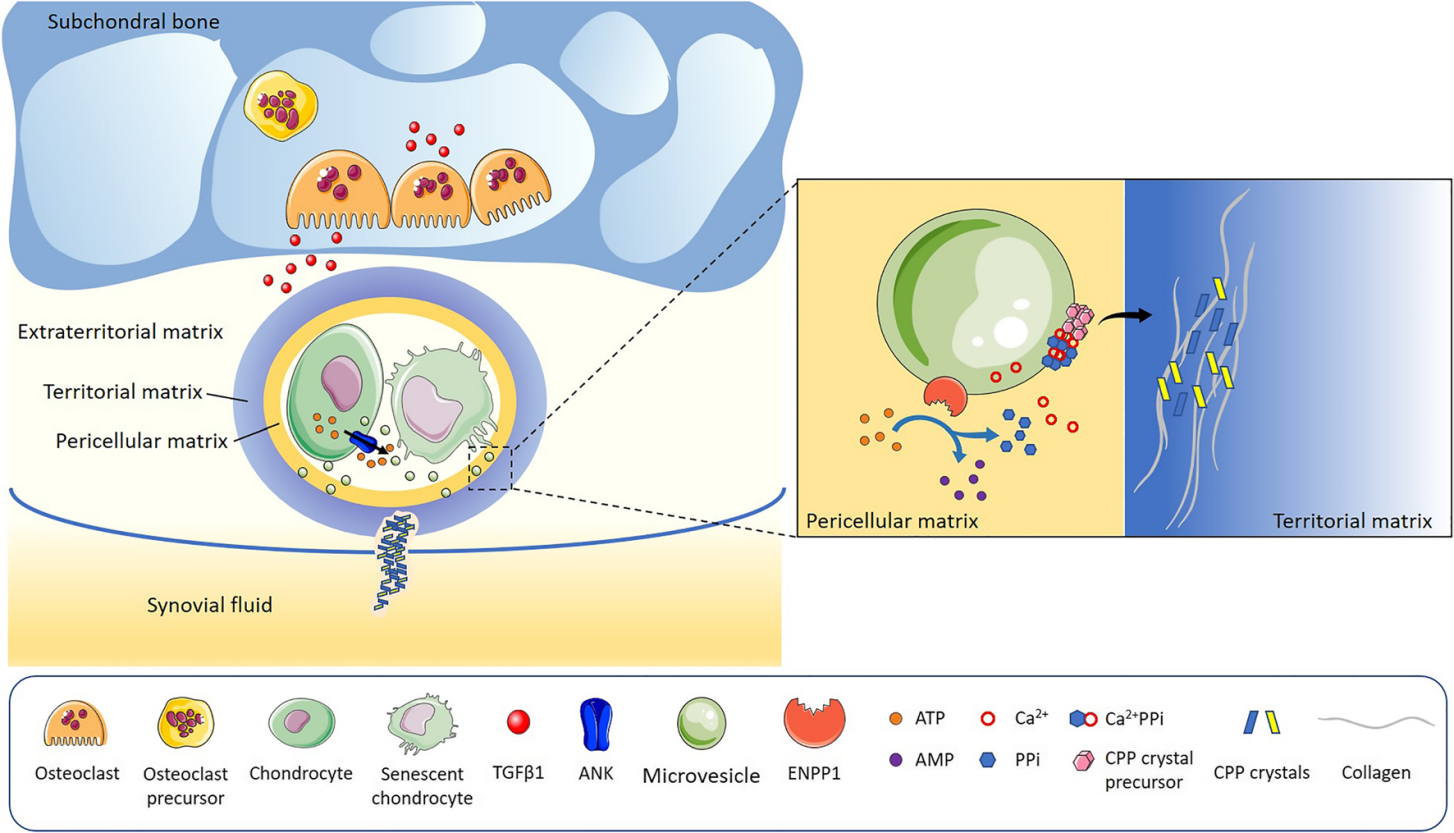
Main mechanisms involved in CPP crystal formation. Activated osteoclasts in subchondral bone release TGFβ1 into cartilage, which induces an increase in the levels of nucleotide pyrophosphohydrolase (NTPPH) enzymes and PPi production. Chondrocytes and senescent chondrocytes release articular cartilage vesicles (ACVs) in pericellular regions of extracellular matrix that contain NTPPH enzymes, particularly ENPP1. In the same cells, Ank mediates the extracellular efflux of ATP. In ACVs, ENPP1 hydrolyzes ATP in PPi and AMP. Excess PPi complexes with calcium to form amorphous CPP precursors, which are then converted into more stable CPP crystals and grow within or along collagen fibrils. Once formed in the ECM, CPP crystals may shed into the synovial fluid. TGFβ1: transforming growth factor beta 1; ANK: progressive ankylosis protein; ENPP1: ectonucleotide pyrophosphatase/phosphodiesterase 1; ATP: adenosine triphosphate; AMP: Adenosine monophosphate; PPi: inorganic pyrophosphate; CPP: calcium pyrophosphate

CPP crystals primarily form within fibrocartilage, hyaline cartilage, or tissue undergoing chondroid metaplasia, with chondrocytes being the principal cells involved in their production [6]. Chondrocytes represent the sole cell type in articular cartilage (comprising both hyaline cartilage and fibrocartilage) and contribute to CPP crystal formation by producing high levels of extracellular inorganic pyrophosphate (PPi) and generating articular cartilage vesicles (ACVs) [7]. It has been reported that ACVs are mainly derived from hypertrophic chondrocytes, however, a recent study indicates that CPP crystal deposition is associated with aging and chondrocyte senescence. Indeed, cellular senescence markers, such as p16 and p21, are increased in CPPD cartilage compared to OA cartilage [8] (Fig. 1).

While it's intuitive to attribute CPP crystal formation to cartilaginous structures, the presence of these crystals in non-cartilaginous tissues like tendons and ligaments, where chondrocytes are typically absent, raises intriguing questions. This could be explained by the fact that the enthesis of tendons and ligaments presents a fibrocartilaginous layer [9], thus providing a plausible explanation for the preferential deposition of CPP crystals at the insertions of tendons and ligaments. Additionally, changes in extracellular matrix composition and the presence of chondrocyte-like cells, instead of fibroblasts, have been observed around CPP and basic calcium phosphate (BCP) deposits in tendons [10, 11]. The acquisition of chondrocyte phenotype and the consistent formation of calcific deposits in the midsubstance of tendons could be due to an erroneous differentiation of tenocytes into fibrocartilage, or more likely to a fibrochondrogenic differentiation of human tendon-derived stem/progenitor cells [11].

CPP crystals can also be found in the synovial membrane, but it remains uncertain whether they are produced by synoviocytes or derived from synovial fluid (SF) and articular cartilage [5]. Nonetheless, it is recognized that CPP crystal contributes to synovitis by stimulating synovial fibroblast to increase the production of matrix metalloproteinase-8 (MMP-8) and Interleukin 6 (IL-6) [12, 13]. Macrophages are the primary inflammatory cells in the synovial membrane and are implicated in promoting inflammation and contributing to joint damage [5]. In vitro studies have shown that upon stimulation by CPP or BCP crystals, macrophages release pro-inflammatory cytokines and chemokines, exacerbating inflammation and stimulating the production of reactive oxygen species (ROS) and matrix-degrading enzymes by chondrocytes [14, 15]. Furthermore, macrophages' attempted digestion of insoluble crystals often results in inefficient phagocytosis, leading to lysosomal rupture, ROS production, adenosine triphosphate (ATP) release and potassium efflux, activating the NACHT domain-, leucine-rich repeat-, and PYD-containing protein 3 (NLRP3) inflammasome [16], resulting in the production of IL-1β, which perpetuates inflammation through additional upregulation of cytokines and chemokines, neutrophil recruitment, and production of inflammatory mediators [15, 17].

In addition, CPP crystals have been found to trigger a regulated form of cell-death, known as necroptosis, in fibroblasts, epithelial cells, and neutrophils [16]. This process is linked with the formation of neutrophil extracellular traps (NETs), that contribute to inflammation. All these mechanisms, which activate the innate and adaptive immune system, can explain the acute or chronic arthritis phenotypes observed in CPPD, and moreover, low-grade synovitis can be linked to the progression of joint damage.

Bone health and repair processes have been reported to play a key role in CPPD [18]. Osteoclasts in subchondral bone seem to be a new player in the CPP crystal formation scenario, and this is related to an excess of osteoclast activity, which may release transforming growth factor beta (TGFβ) and other factors into cartilage, increasing PPi production [7, 18]. The increased presence of osteoclasts in the subchondral bone of humans and mice with OA, along with aberrant bone structures and reduced mechanical strength, supports this hypothesis. CPP crystals enhance osteoclastogenesis mediated by receptor activator of nuclear factor-κB ligand (RANKL) and macrophage colony-stimulating factor 1 (M-CSF) by promoting the p38 mitogen-activated protein kinase (MAPK) and extracellular-signal-regulated kinase (ERK) pathways [19].

### Extracellular Matrix and Synovial Fluid (SF) Changes in CPPD

Histologic and in vitro observations suggest that CPP crystals form extracellularly, initially in ACVs in pericellular regions of extracellular matrix [20–22], where excess PPi can accumulate and complex with calcium. ACVs are chondrocyte-derived organelles that contain enzymes involved in both CPP and BCP crystals formation [23–25]. Among these enzymes, nucleotide pyrophosphohydrolases (NTPPH) play a key role in extracellular PPi production by hydrolyzing ATP [7]. In particular, the main participant was identified in NTPPH enzyme plasma­cell membrane glycoprotein 1 (ENPP1/PC­1), a regulator of tissue mineralization. ENPP1 up-regulated expression was detected in calcified degenerative menisci, and associated with intracellular and extracellular PPi (ePPi) [26]. In contrast, ENPP1 downregulation was reported in human and mouse OA cartilage, correlating with increased cartilage calcification [27]. However, direct evidence linking the presence of CPP and ENPP1 levels is lacking in both studies, thus suggesting the need for further investigation into the role of this enzyme and the potential involvement of other factors in these processes. In this context, reduction in tissue-non-specific alkaline phosphatase (TNAP) levels or increase in progressive ankylosis protein homolog (Ank) activity are supposed to regulate ePPi elaboration. TNAP antagonizes ENPP1 by hydrolyzing ePPi to extracellular inorganic phosphate (ePi), thus promoting BCP crystal formation [28, 29]. Ank is a transmembrane transporter detected in high levels in CPPD cartilage [30], whose mutations in the gene encoding are associated with familial CPPD [31, 32]. It has long been debated whether Ank transports PPi or ATP; with recent findings indicating its role in mediating the cellular efflux of ATP rather than PPi [33, 34].

The ePPi levels and enzyme activity are regulated by different factors, such as TGFβ, insulin-like growth factor-1, IL-1β, retinoic acid and thyroid hormone [7]. Usually, increases in ePPi levels correspond to decreases in those of ePi and vice versa, suggesting that Pi/PPi ratio in ACVs is determinant to the type of crystal formed [35]. An imbalance toward PPi production promotes CPP crystal formation, while BCP crystal formation is prevented [36]. Initially, excess PPi complexes with calcium to form amorphous CPP precursors, which are then converted into more stable crystalline forms and grow within or along collagen fibrils [5]. Despite its potential importance, no studies have been conducted on how calcium levels contribute to crystal formation.

CPP crystal formation and growth can also be promoted by other extracellular matrix components, such as osteopontin, transglutaminase and type I collagen [37–39]. In contrast, proteoglycans and type II collagen have been shown to inhibit CPP formation [39]. Interestingly, enhanced type X collagen expression has been observed in CPPD cartilage [40].

Once formed in the extracellular matrix, CPP crystals may shed into the SF. Most studies evaluating the role of SF in crystal formation are dated, and updated investigations are needed to better characterize changes in its composition. In these studies, CPPD patients have higher SF NTPPH, ATP and PPi levels than patients with other arthropathies [41–44]. In addition, it is suggested that high ePPi concentrations in SF are mainly produced by fibrocartilage and hyaline cartilage and are a key factor for CPP crystal formation [32, 45]. However, the effective role and origin of these ions in SF are not yet completely understood and could also derive from the crystal dissolution in this medium. In contrast, a more recent study comparing SF of patients with OA to those with CPPD plus OA, found no significant differences in the concentrations of calcium ions, magnesium ions, Pi and PPi [46].

## Lessons from the Clinics

### CPPD and Association with Other Diseases

Together with OA, CPPD is the most common abnormality observed in joints in older people. Prevalence of CPPD estimated from radiographs of the appendicular skeleton is up to 13.7% at around 60 years of age, and continuously increases with age-groups to reach 50% among people older than 80 years, with no predilection for either sex [6, 16, 47, 48]. While CPPD disease covers a variety of phenotypes ranging from acute inflammatory episodes, persistent CPP crystal inflammatory arthritis, to chronic non-inflammatory joint pain in the context of CPPD associated with OA [2, 6, 49–51], to date, no relationship has been established between the extent and anatomical localization of CPPD and the phenotype(s) of CPPD disease that will eventually develop.

The very specific and rare clinical situations of familial CPPD disease allowed to identify genetic mutations associated with early and severe CPPD, and provided a better understanding of CPP crystal formation in general [32]. Two genes in particular have been linked to these familial phenotypes of CPPD disease: gain-of-function mutations of Ank human gene (*ANKH)* which codes for a transporter of ATP which will be transformed in ePPi, which will precipitate with calcium ions [34, 52], and *TNFRSF11B* which encodes osteoprotegerin [53]. The implication of *ANKH*-associated mutations appears to be responsible for very early onset of CPPD and secondarily of severe joint damage, while people with osteoprotegerin mutations present with a predominant phenotype of early onset of severe and diffuse OA and concomitant CPPD, possibly due to a mechanism involving subchondral bone abnormalities [54]. *ANKH* mutations have also been suggested to be involved in sporadic CPPD disease [55]. However, the preliminary results of the first attempt to inhibit *ANKH* in the clinical setting using probenecid, an anion transporter inhibitor [56], did not show a meaningful clinical impact.

The identification of risk factors for CPPD and associated comorbidities also helped out in the understanding of the pathophysiology of CPPD, and on the general balance between CPPD and hydroxyapatite, involved in bone formation. Gitelman’s disease, a renal tubulopathy characterized by severe hypomagnesemia, has been linked with severe and extensive CPPD, including involvement of the axial skeleton [57]. Magnesium is a natural inhibitor of TNAP, which cleaves PPi into Pi, thereby shifting the equilibrium from CPP crystal formation towards hydroxyapatite [34]. While the only four-decade-old trial including 38 patients using magnesium supplementation failed to demonstrate a decrease in the extent of CPPD on radiographs at 6 months, its results suggested that magnesium could have a positive impact on pain relief [58].

The recently proposed association between CPPD disease and osteoporotic fractures may bring new insight into the balance between CPP crystal and hydroxyapatite formation [59]. In a study including 1148 patients with acute CPP crystal arthritis matched to 3730 comparators, the fracture relative risk was found to be twice as high in participants with CPPD (hazard ratio 1.8 [95% confidence interval 1.3–2.3]) [59]. Given that TNAP plays a central role in the balance between PPi and Pi, and sporadic CPPD disease appears to be associated with osteoporosis, it is probable that TNAP regulation needs to be further explored to understand this association. Moreover, this association may be more complex than the sole imbalance in calcium crystal formation and also involves the role of systemic inflammation, suspected to be responsible for the increased risk of cardiovascular events following acute episodes of CPP crystal arthritis [60, 61], as it has been shown in gout [62].

### Imaging Contribution in CPPD Understanding

The link between CPPD and imaging extends to 1960, even before CPP crystals identification in SF, when the calcification of articular cartilage, termed “chondrocalcinosis” was first documented by Zitnan and Sitaj [63]. Since then, imaging has contributed to characterizing the disease, assessing the prevalence of calcification in joint structures, elucidating the association of CPPD with other conditions and facilitating differential diagnosis.

**Fig. 2:**
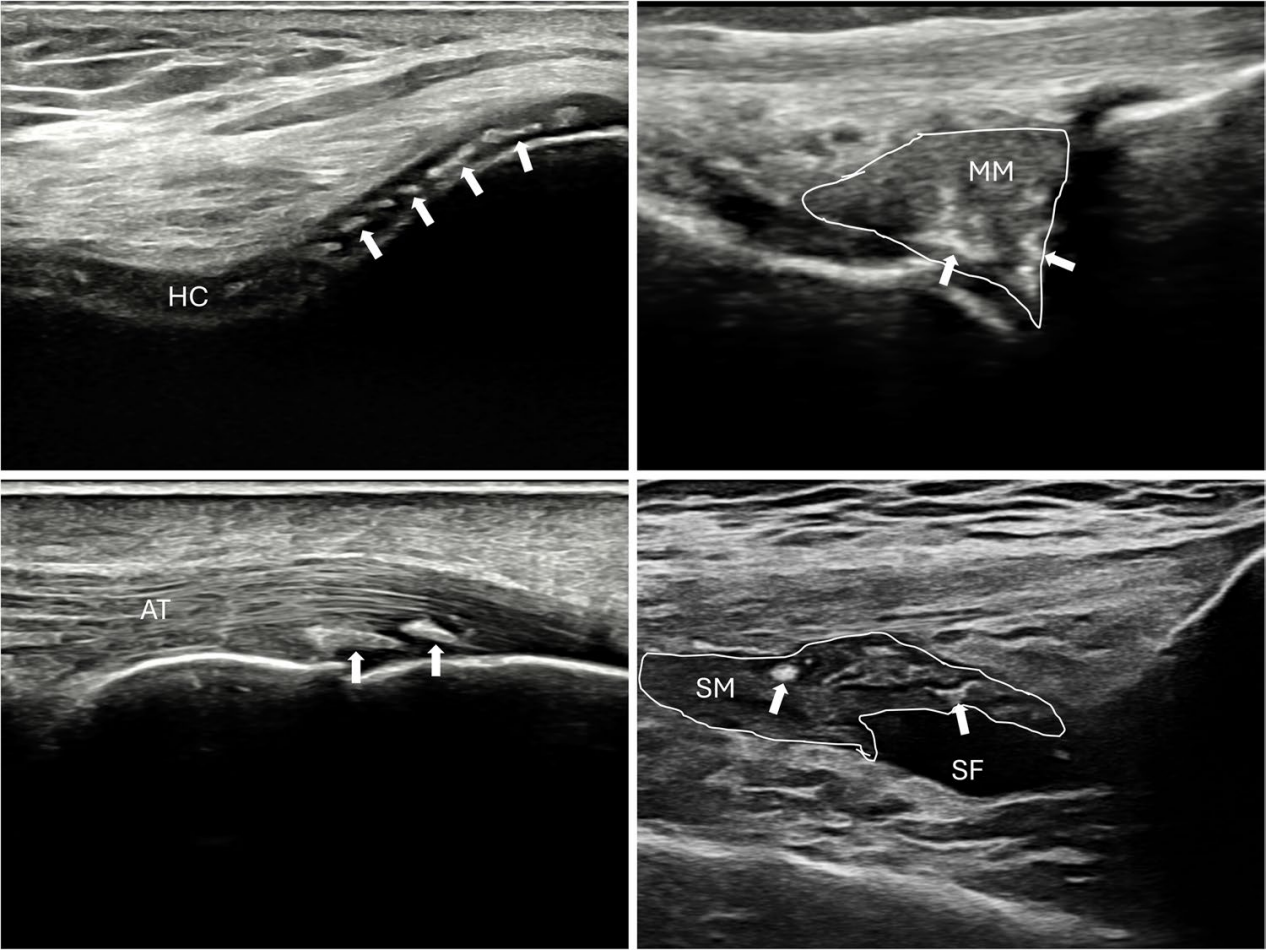
Ultrasonographic appearance of CPPD in various joint structures. CPP deposits (arrows) appear as hyperechoic (white) deposits (similar to the bone cortex) without creating acoustic shadowing. In fibrocartilage, hyaline cartilage, and synovial. membrane, they present as deposits of variable size and shape, while in tendons, they appear as multiple linear deposits parallel to the tendon’s fibres, not continuous with the bone profile. A) CPP deposits (arrows) in femoral hyaline cartilage, B) in fibrocartilage (knee meniscus traced with a white continuous line), C) in tendons (Achilles tendon), and D) in the synovial membrane (synovial hypertrophy traced with a white continuous line). HC: hyaline cartilage, MM: medial meniscus, AT: Achilles tendon, SM: synovial membrane, SF: synovial fluid

Imaging techniques have played a crucial role in defining CPPD as a systemic disease affecting both peripheral and axial regions [49, 64]. They have also allowed us to understand that CPP crystal deposition is not confined to the hyaline cartilage, as initially thought, but extends to other joint and periarticular structures, including fibrocartilage, tendons, ligaments and synovial membrane [65–68] (Fig. 2). A radiological study conducted at the knee level by Neame et al., demonstrated that the fibrocartilage was the most affected structure in CPPD, observed in 95% of cases, compared to hyaline cartilage in 45% of cases, and joint capsules or the synovial membrane in 30% of cases [69]. These findings were corroborated by a recent systematic literature review (SLR) assessing the prevalence of CPPD across various joint structures using ultrasound and radiography [70] (Table 1). The SLR revealed a higher prevalence in the knee menisci and the triangular fibrocartilage of the wrist, compared to the knee hyaline cartilage. Despite such evidence, much of the basic research has remained narrowly focused on hyaline cartilage. However, shifting perspectives may provide a pathway to delve deeper into understanding this complex disease.

**Table 1:** Prevalence and distribution of CPPD in various joint structures in patients with CPPD disease. This table summarizes findings from a systematic literature review (SLR) that evaluated the prevalence of CPPD using conventional radiography (CR) and ultrasound (US) [70], in comparison to findings on CPPD prevalence of an ultrasonographic study [105]. For the SLR, we report the maximum prevalence of CPPD detected by CR or US, with the method used for CPPD identification indicated in parentheses. The differences in the results arise because US is more sensitive than CR in CPPD detection. FC: fibrocartilage, HC: hyaline cartilage, TFCC: triangular fibrocartilage complex

	Adinolfi et al. [70]	Cipolletta et al. [105]
Knee	**88% (US)**	**97% (US)**
FC (menisci)	90%	91%
HC	66%	65%
Tendons	NA	36%
Wrist	**92% (US)**	**80% (US)**
FC (TFCC)	56%	96%
Ligaments	NA	63%
Hip	**65% (CR)**	**56% (US)**
FC	39%	96%
HC	26%	26%
Elbow	**44% (CR)**	**66% (US)**
HC	32%	43%
Tendons	11%	62%
Shoulder	**31% (CR)**	**63% (US)**
FC	4%	31%
HC	11%	20%
Tendons	7%	NA
Acromion-clavicular	**42% (CR)**	**58% (US)**
Hand (HC)	**19% (CR)**	**24% (US)**
Ankle	**17% (CR)**	**46% (US)**
HC	19%	25%
Tendons	%	83%

Moreover, imaging has shed some light on how CPP crystals deposit within joint structures. The identification of the pseudo double contour sign on ultrasound or cartilage icing on radiography has enhanced our understanding, indicating CPP crystal deposition not only within but also over the cartilage [71–73]. Though the exact mechanism remains unclear, a recent study offers an anatomopathological explanation of the pseudo double contour sing, suggesting CPP deposits within the capsule/ligament atop the hyaline cartilage [71]. Thus, dynamic scanning can be useful in distinguishing the pseudo double contour of CPPD from the double contour characteristic of gout [72].

Imaging has also been instrumental in enhancing our understanding of CPPD epidemiology. Radiographic cartilage calcification (formerly known as chondrocalcinosis) has conventionally been considered a surrogate for CPPD prevalence, and radiographic studies have shown CPPD prevalence ranging from 7% to 13.7% at around 60 years of age, increasing with age to 50% among individuals over 80 years old [6, 16, 48, 74]. Ultrasound, with its higher sensitivity, has gone further, revealing CPPD in over 55% of patients presenting at outpatient rheumatologic clinics for the first time with any joint complaint, rising to 66% among those older than 60 and 73% among individuals aged 80 years or older [75]. However, the exact prevalence of CPPD remains undetermined, as current studies have focused solely on symptomatic individuals. Nonetheless, imaging techniques, particularly ultrasound, hold promise for conducting epidemiological studies in the general population.

A lesser-explored aspect of CPPD is its natural history. In this realm, imaging techniques, particularly ultrasound, thanks to the recently developed ultrasonographic scoring system for assessing CPPD extent [76], could provide new insight into the evolution of CPP crystal deposition over time, for example following therapy or after acute attacks, as well as exploring the correlation between CPPD extent and joint damage or OA, and moreover can offer a means of monitoring the efficacy of potential therapies.

## The future

### Raman Spectroscopy “for Everyone”

Raman spectroscopy is a technique based on the interaction between light and electrons in a material. Atoms in a molecular bond make oscillatory motions, modulating the electronic response with unique frequencies, enabling precise material identification. When irradiated with monochromatic (laser) light, the small energy changes associated with the modulation of the bonds lead to a distinct presentation of higher and lower frequencies in the scattered light. This can be measured as a color shift from the original light source. Analysis of the color spectrum of the scattered light provides a fingerprint of the bonds present in the sample, useful for identifying the material. CPP crystal is identified by several specific Raman features, most notably a strong band which can be attributed to the symmetric P-O stretching mode [77]. The location of this band is dependent on the type of CPP crystal, and is found at 1049 cm^−1^ for triclinic CPP dihydrate, 1045 cm^−1^ for monoclinic CPP dihydrate, and 1036 cm^−1^ for monoclinic CPP tetrahydrate β [78]. These specific spectral characteristics allow for detailed crystal analysis, revealing even subtle differences in structure. This is interesting as different crystal phases may elicit different inflammatory responses [15].

**Fig. 3:**
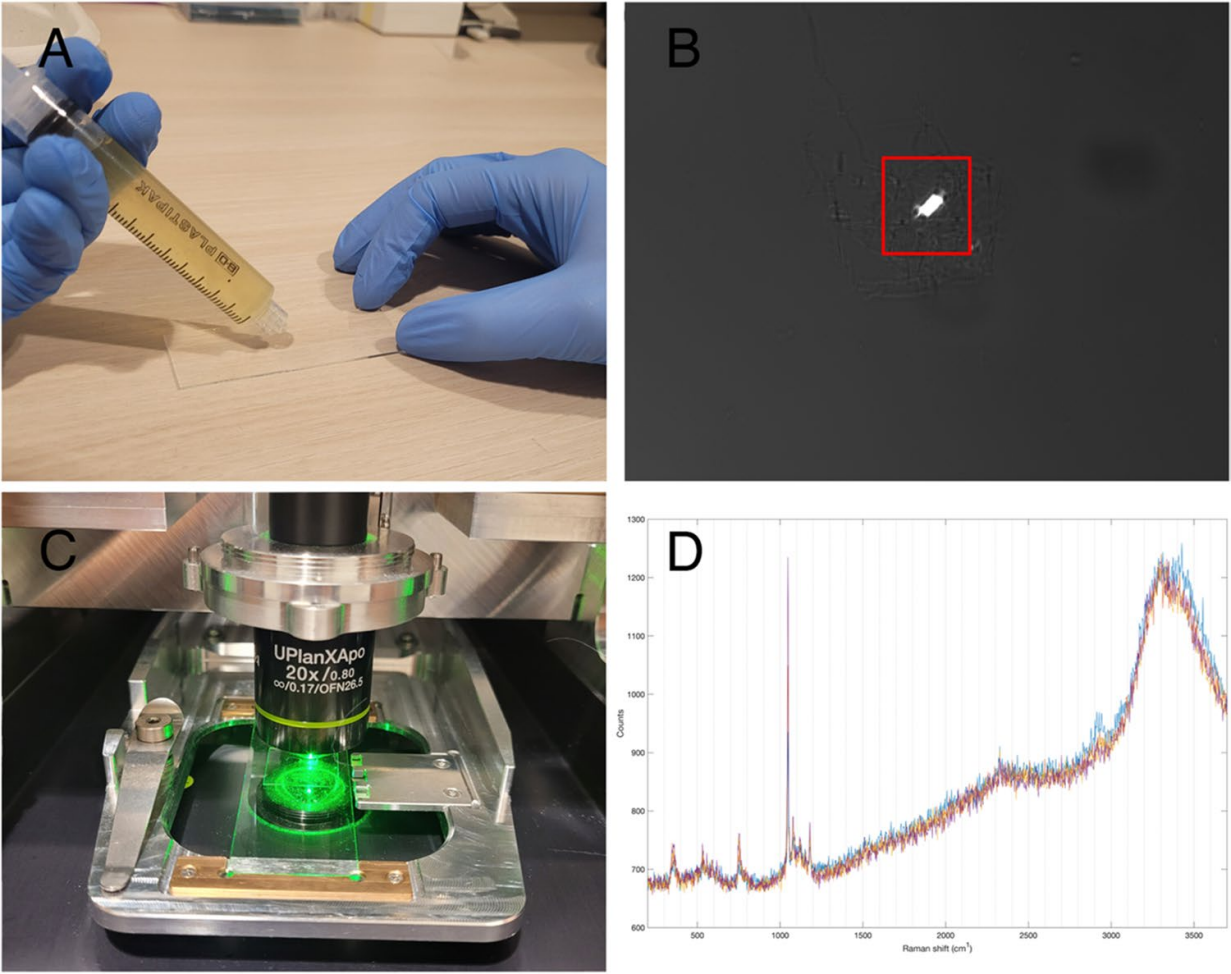
A hybrid combination of hyperspectral Raman imaging and polarized light microscopy can identify CPP with high specificity. A) Raman spectroscopic imaging can be performed with unprocessed synovial fluid, using standard microscope slides. B) Ordinary polarized light microscopy is used to locate birefringent crystals such as CPP or MSU. Once located, an area of interest is determined by the operator. C) Using the integrated Raman spectroscope, selected crystals are automatically scanned. D) The result: a collection of Raman spectra of the triclinic CPP crystal shown in panel B)

The current gold standard for SF crystal analysis is compensated polarized light microscopy (CPLM), which is significantly less accurate for CPP crystals compared to monosodium urate (MSU) crystals [79, 80]. The birefringence of triclinic CPP is roughly 20 × lower than that of MSU [81], and the varied crystal morphology of CPP further complicates its identification with CPLM. Additionally, BCP crystals are even less birefringent and therefore not identifiable with CPLM. Raman spectroscopy can be used to identify CPP, BCP, but also calcium oxalate crystals in SF and tissue with 100% spectral specificity. Spectroscopic techniques are more objective than operator-dependent microscopic classification and are also well-suited for artificial intelligence-based classification models [82]. In SF analysis, Raman spectroscopy is not performed on bulk material; instead, laser light must be focused directly on the crystals, which can be challenging due to their small size and relative sparsity. Li et al. developed a point-of-care Raman spectroscope capable of isolating and analyzing CPP crystals from SF using filtration and enzymatic digestion [83]. While effective, this method is also time-consuming and destructive to the sample. Raman hyperspectral imaging, however, can analyze crystals directly in SF, but requires manual crystal selection. Adding polarization filters to a hyperspectral Raman microscope has proven helpful and has been successfully implemented [84] (Fig. 3). In a consecutive series of 400 SF samples, this method demonstrated an 86.0% sensitivity and 99.1% specificity for CPP crystal identification according to the 2023 ACR/EULAR CPPD disease classification criteria [85]. Additionally, hyperspectral Raman imaging can be applied to identifying CPPD in tissue slides [86]. With a spatial resolution of around 1 μm, Raman imaging can provide detailed information on the nature and morphology of CPP foci in ex vivo tissue.

Future applications of Raman spectroscopy in rheumatology include the study of calcium crystals in OA [87] and the investigation of the role of body-foreign material, such as nanoparticles and microplastics, in inflammatory joint disease [88, 89]. Raman spectroscopic examination of SF from OA patients demonstrated the presence of BCP and CPP crystals, as well as calcium oxalate crystals and a novel crystal type: calcium carbonate [87]. All of these crystals exhibit pro-inflammatory properties, suggesting they might play a role in OA progression. Body foreign materials can also be pro-inflammatory and might cause joint inflammation. Raman spectroscopic identification of the causative particles might provide insights into new disease pathways and has already been demonstrated in clinical practice.

A series of validation studies have shown clear clinical benefits of using Raman spectroscopy for diagnosing CPPD. Depending on the type of Raman spectroscope, analysis takes between 5 to 15 min. While Raman spectroscopes are more expensive than CPLM, typically costing between €100,000 and €400,000, their rapid processing times allow to analyze thousands of SF samples annually. The design of Raman devices dedicated for clinical use will reduce the amount of training required for operating the machine. Overall, the implementation of Raman spectroscopy in CPPD assessment could enhance the specificity, objectivity, and patient stratification.

### New Approaches in Basic Science

New laboratory approaches in the study of CPPD, and particularly those related to omics sciences, have seen limited application thus far. The omics path begins with the analysis of the gene sequences and genomics, and proceeds towards the investigation of the transcription profiles, transcriptomics, and the protein expression profile, proteomics, up to the functional results of proteins activity, metabolomics.

In the last twenty years, genetic studies, first, and genomics approaches, subsequently, have identified loci associated with abnormal cartilage calcifications. Familial cases of CPPD appear inherited in an autosomal dominant manner. Monogenic forms have been associated with mutations in genes determining the increase of ePPi as loss-of-function mutations in *ANKH* gene [32, 90], encoding for Inorganic Pyrophosphate Transport Regulator, *PC-1*, encoding for a nucleotide pyrophosphate synthetase [32], *TNAP*, encoding for tissue-non-specific alkaline phosphatase found in matrix vesicles and also associated with hypophosphatasia [91, 92]. However, later studies have not consistently confirmed some of these associations, as in the case of *TNAP* and *ENPP1*, encoding for ectonucleotide pyrophosphatase [93]. Associations have also been noted with genetically determined iron and copper metabolism disorders (haemochromatosis, Wilson’s disease), as well as phosphate disorders (X-linked dominant hypophosphataemic rickets due to mutations in the *PHEX* gene) [91, 92]. A 2017 whole-genome-wide linkage study failed to identify specific gene variants associated with CPPD, including those in *ANKH*, in ten individuals from five pedigrees [94]. Recently, a whole exosome sequencing of patients with diffuse idiopathic skeletal hyperostosis and CPPD suggests an association with variants of a new gene, *PPP2R2D*. This gene encodes a serine/threonine protein phosphatase that regulates basal cellular activities by dephosphorylating substrates. [95]. Ectopic calcifications have been observed in mice carrying a gain-of-function mutation in the *TNFRSF11B* gene, which encodes for osteoprotegerin, an osteoblast-derived inhibitor of the pro-osteoclastogenic RANK-RANKL axis [54, 96]. Further, read through mutation in *TNFRSF11B* results in the translation of an aberrant protein with additional 19 amino acids at its C-terminus (OPG-XL), leading to excessive fibrosis and mineralization in cartilage [97].

To our knowledge, no transcriptomic studies have been conducted on CPPD. However, in the few published articles analysing the expression profiles of chondrocytes isolated from calcium-containing crystal cartilages, a common finding was the upregulation of the expression of genes involved in mineralization, such as *ANKH*, *PC-1* and *TNAP* [98]. Interestingly, in human primary articular chondrocytes, in vitro, the histone deacetylase inhibitors (HDACi), trichostatin A and vorinostat, downregulate mRNA and protein expression of *ANKH* and *ENPP1* and upregulate *TNAP*, limiting levels of ePPi [99].

In addition, there is a notable absence of proteomics studies in this area. In 2020, de Seny et al. attempted to delineate common inflammatory mechanisms among synovial membranes affected by OA, CPPD and rheumatoid arthritis (RA). Among the 4336 proteins identified by mass spectrometry, 51 were selected for their strong correlation with histological scores. Of these, 11 proteins (DNAJB11, CALR, ERP29, GANAB, HSP90B1, HSPA1A, HSPA5, HYOU1, LMAN1, PDIA4, and TXNDC5) are involved in the endoplasmic reticulum stress. S100A8 and S100A9 were significantly elevated in RA compared to OA (both) or CPPD (S100A8 only) and significantly correlated with histological scores [100]. Further, 1871 proteins were associated with histological inflammatory scores and, of these, 10 proteins (LAP3, MANF, LCP1, CTSZ, PTPRC, DNAJB11, EML4, SCARA5, EIF3K, C1orf123) resulted differentially expressed in the synovial membrane of at least one of the three disease groups [101].

To the best of our knowledge, no metabolomics studies are available in the field of CPPD. The synovial joint is a closed system with limited exchange with the external environment, such as the bloodstream. Therefore, besides genomics, omics studies should highlight the similarities in the two environments associated with the disease. Another approach could rely on the spatial analysis of variations in cellular and molecular structures in the target tissue, surely providing a more detailed understanding of the pathophysiology. Spatially resolved biology enables the investigation of cells within the context of their tissue microenvironment. These techniques allow for genomics, transcriptomics, proteomics, and metabolomics analysis of intact tissue sections, associated with precise spatial coordinates [102].

## Conclusions

The journey toward a comprehensive understanding of CPPD formation and deposition is ongoing. Progress has been made through basic science research, imaging studies, and investigations into the genetic forms of CPPD, yet significant gaps in knowledge remain. Emerging techniques in both imaging and basic sciences offer promise for deepening our understanding of this complex disease.

This is an exciting time for CPPD research, supported by international initiatives that lay the groundwork for standardized approaches to the disease. Notable advancements include the ACR/EULAR’s first set of classification criteria for CPPD disease [49], the OMERACT US definitions and scoring system [65, 66, 74, 76], the ACR/EULAR recommendations for the use of imaging in crystal-induced arthritis [103], the G-CAN project refining CPPD nomenclature, and the OMERACT project establishing a core outcome domain set for CPPD [104]. With current technological advancements and increasing scientific knowledge, the potential for breakthroughs has never been greater, and this is the perfect time to embark on new research initiatives.

## Data Availability

No datasets were generated or analysed during the current study.
